# Long non-coding RNA Malat1 activated autophagy, hence promoting cell proliferation and inhibiting apoptosis by sponging miR-101 in colorectal cancer

**DOI:** 10.1186/s11658-019-0175-8

**Published:** 2019-07-27

**Authors:** Yaoran Si, Zhaoguo Yang, Quanxing Ge, Lingbing Yu, Meiying Yao, Xinfang Sun, Zheng Ren, Chunsheng Ding

**Affiliations:** 1 Department of Gastroenterology, Huaihe Hospital, Henan University, Kaifeng, 475000 Henan China; 2 Department of General Surgery, Kaifeng Central Hospital, Kaifeng, Henan China

**Keywords:** Colorectal cancer, Long noncoding RNA Malat1, Autophagy, Proliferation, Apoptosis, miR-101

## Abstract

**Background:**

Long non-coding RNA Malat1 has been widely identified as an oncogene which shows a significant relationship with tumorigenesis in colorectal cancer (CRC). Nonetheless, whether Malat1 participates in the autophagy of colorectal cancer remains unclear.

**Materials and methods:**

First, the expression level of Malat1 in 96 pairs of colorectal cancer tissues and four cell lines was detected by qRT-PCR. Subsequently, the autophagy activity in colorectal cancer tissues and cell lines was detected by western blot. Furthermore, the CCK-8 assay and flow cytometry (FCM) were performed to detect the role of autophagy activated by Malat1 in colorectal cancer cell lines.

**Results:**

In this study, significantly increased Malat1 expression and autophagy activity were found in colorectal cancer tissues compared with the adjacent normal tissues. Also, the Malat1 level was positively correlated with the expression of LC3-II mRNA in vivo. Moreover, autophagy activation and cell proliferation were significantly facilitated by Malat1 in colorectal cancer cells, while apoptosis decreased. Above all, the inhibition of autophagy by 3-MA not only relieved the Malat1-induced cell proliferation but also promoted the Malat1-induced cell apoptosis. In addition, Malat1 was found to act as an endogenous sponge by directly binding to miR-101 to reduce miR-101. Furthermore, the suppressive effects of miR-101 on the autophagy, proliferation, and apoptosis of CRC were abolished by Malat1.

**Conclusion:**

Long non-coding RNA Malat1 activated autophagy and promoted cell proliferation, yet inhibited apoptosis by sponging miR-101 in colorectal cancer cells.

## Introduction

Long non-coding RNA (LncRNAs) and non-coding RNAs longer than 200 nucleotides [1, 2] function by interacting and regulating various types of genes and proteins via diverse mechanisms [3], thereby participating in a variety of fundamental physiopathologic processes, such as carcinogenesis, autophagy, cardiovascular and neurological diseases [4–6]. In addition, lncRNAs have been revealed to function as competing endogenous RNAs (ceRNAs), which can sequester the common microRNAs (miRNAs) and thereby prevent the miRNAs binding to their ancestral gene [7].

Recently, many studies have indicated that lncRNAs can interact with several autophagy-related genes at different stages to regulate autophagy [8]. Metastasis-associated lung adenocarcinoma transcript 1 (Malat1), as a member of lncRNAs, is highly conserved among mammals and is strongly expressed in the nucleus [9]. Increasing reports have demonstrated that Malat1 is highly expressed in different types of cancer patients and has a strong relationship with the prognosis of cancer patients [10].

Autophagy, widely known as macroautophagy, can be characterized by delivering cytoplasm components, which can be enclosed in double-membrane vesicles, to lysosomes for degradation [11]. Thereby, autophagy is crucial in a variety of pathological and physiological processes, particularly malignant tumor progression [12]. Recently, numerous studies have demonstrated that as a self-protective mechanism, autophagy can be regulated by lncRNA in cancer cells. Wang Y et al. found that BANCR not only contributes to cell proliferation but also activates autophagy in papillary thyroid carcinoma [12]. Yang L et al. indicated that the long noncoding RNA HOTAIR, through interaction with ATG3 and ATG7, can activate autophagy in hepatocellular carcinoma [13]. Also, increasing reports have indicated that Malat1 activates autophagy and participates in tumorigenesis, such as cell proliferation, apoptosis and metastasis, in a number of cancer cells [9, 14–18]. Nonetheless, rare reports have focused on the molecular mechanism of Malat1 on autophagy in CRC.

In this paper, quantitative real-time PCR (qRT-PCR) was performed to detect the expression level of Malat1 in CRC tissues and cell lines. The association between Malat1 expression and CRC cell autophagy, proliferation and apoptosis was also investigated to evaluate the role of Malat1 in CRC. Furthermore, this study explored the molecular mechanism whereby Malat1 exerted regulatory effects on CRC cell autophagy, proliferation and apoptosis.

## Materials and methods

### Patients and collection of clinical samples

Ninety-six colorectal cancer tissues and paired non-cancer tissues were obtained from the surgery carried out at the Huaihe Hospital of Henan University from May 2012 to November 2016. These tissues were stored in liquid nitrogen. The present study was approved by the Ethics Committee of Henan University (Henan, China) and all the patients signed the informed consent before the examination.

### Cell cultures

Normal human colon epithelial cell line FHC and 4 colorectal cancer cell lines (HT29, HCT116, SW480, SW620) were purchased from the American Type Culture Collection (USA) and cultured in the DMEM Medium, McCoy’s 5a Medium, and Leibovitz’s L-15 Medium (Gibco BRL, Gaithersburg MD) with 10% fetal bovine serum, as well as cells cultured in the humidified atmosphere of 95% air and 5% CO_2_ at 37 °C. For the in vitro assay, to reveal the effect of Malat1 on autophagy, the cells were treated with 3-MA (3-methyladenine) [19].

### RNA extraction and the quantitative real-time PCR

According to the manufacturer’s instructions, the total RNAs extracted from tissues and cells were isolated from the Trizol reagent (Invitrogen, Grand Island, CA, USA). The isolated RNAs were first reversely transcribed to cDNA with the PrimeScript RT reagent Kit (Takara, Japan) following the manufacturer’s protocol. qRT-PCR was performed with the SYBR Prime Script RT-PCR Kits (Takara, Japan) based on the manufacturer’s protocol. The primers were as follows: MALAT1, 5′-AATGTTAAGAGAAGCCCAGGG-3′ (forward), 5′-AAGGTCAAGAGAAGTGTCAGC-3′ (reverse); GADPH 5′-GCATCCTGGGCTACACTG-3′ (forward), 5′-TGGTCGTTGAGGGCAAT-3′ (reverse); miR-101: 5′-GAGGGGTACAGTACTGTGATA-3′ (forward), 5′-TGCGTGTCGTGGAGTC-3′; U6, 5′-CTCGCTTCGGCAGCACA-3′ (forward), and 5′-AACGCTTCACGAATTTGCGT-3′ (reverse). All the assays were performed in triplicate. The relative expression levels were first calculated using the 2^-ΔΔCt^ method and then normalized to the expression of GAPDH mRNA.

### Cell transfection

The plasmid complementary DNA Malat1 and miR-101 were constructed by the amplification and introduction of Malat1 and the miR-101 cDNA sequence into the pcDNA vector (ABM, Canada). The siRNA sequences targeting Malat1 (si-Malat1) and control (si-RNA) were purchased from Genepharma Co., Ltd. (Shanghai). si-Malat1: 5′-CACAGGGAAAGCGAGTGGTTGGTAA-3′. si-RNA: 5′-UUCUCCGAACGUGUCACGUTT-3′. Both the miR-101 mimics (miR-101) and control (miR-control) were purchased from Bioneer Corp. (Daejeon, Korea). According to the manufacturer’s protocol, the Lipofectamine 2000 kit (Invitrogen) was employed to perform cell transfection. Simply, after being cultured in the 24-well plate, HCT116 and SW620 were transfected with the ratio of si-Malat1/si-NC to transfection reagent (1 μg: 5 μL) and the ratio of pcDNA-Malat1/pcDNA-miR101/pcDNA to transfection reagent (1:4). The mixture was maintained at room temperature for 10–15 min. After aspirating the medium from the plate and washing it once with PBS or serum-free medium, the cells were incubated for 48 h and then used in the subsequent experiments.

### Western blot analysis

After being separated by 10% sodium dodecyl sulfate polyacrylamide gel electrophoresis (SDS-PAGE), cell protein lysates were first transferred to polyvinylidene fluoride membranes (Roche), and later incubated with the specific rabbit anti-human antibodies (Abcam, Shanghai), including LC3-I (ab51520, 1: 5000 dilution), LC3-II (ab51520, 1: 5000 dilution), P62/SQSTM1 (ab91526, 1: 5000 dilution), cleaved caspase-3 (ab32042, 1: 5000 dilution), cleaved caspase-9 (ab2324, 1: 5000 dilution), and β-actin (ab8227, 1: 3000 dilution). Subsequently, they were stored overnight at 4 °C, followed by treatment with secondary anti-rabbit antibodies (A32732, 1:1000 dilution, Thermo Fisher Scientific, American), where the ECL chromogenic substrate was applied in the quantification by densitometry (Quantity One software; Bio-Rad, Hercules, CA, USA).

### Cell proliferation assay

The CCK-8 kit (Dojindo Laboratories, Kumamoto, Japan) was utilized to assess the viability of the cells, which were later seeded in a 96-well plate at a density of 1 × 10^4^ cells per well. After being cultured for 24 h, the corresponding Malat1 and siRNA were transfected and cultured in normal media. After adding the CCK-8 solution at 0 h, 24 h, 48 h and 72 h, the relative number of cells was evaluated at OD 450 nm. All the assays were performed in triplicate.

### Cell apoptosis assay

According to the manufacturer’s instructions, the cells were washed with PBS, and apoptosis was performed using flow cytometric analyses with Annexin V: 7-AAD Apoptosis Detection Kits (BD Biosciences, USA). After incubation, the samples were analyzed using flow cytometry (FACSCalibur, BD Biosciences, San Jose, CA). All the samples were assayed in triplicate.

### Statistical analysis

The SPSS 20.0 software (SPSS Inc., Chicago, IL) was used to conduct all the statistical analyses in this study. Student’s t-test was conducted to compare the two groups and a one-way ANOVA or χ^2^ test was used to analyze the multiple group comparisons. Spearman’s correlation analysis was adopted to detect the correlation between Malat1 and LC3-II/miR-101 expression levels in the CRC tissues, where *P* < 0.05 was considered statistically significant.

## Results

### Malat1 was remarkably overexpressed in CRC, and associated with autophagy activation in CRC

**Fig. 1:**
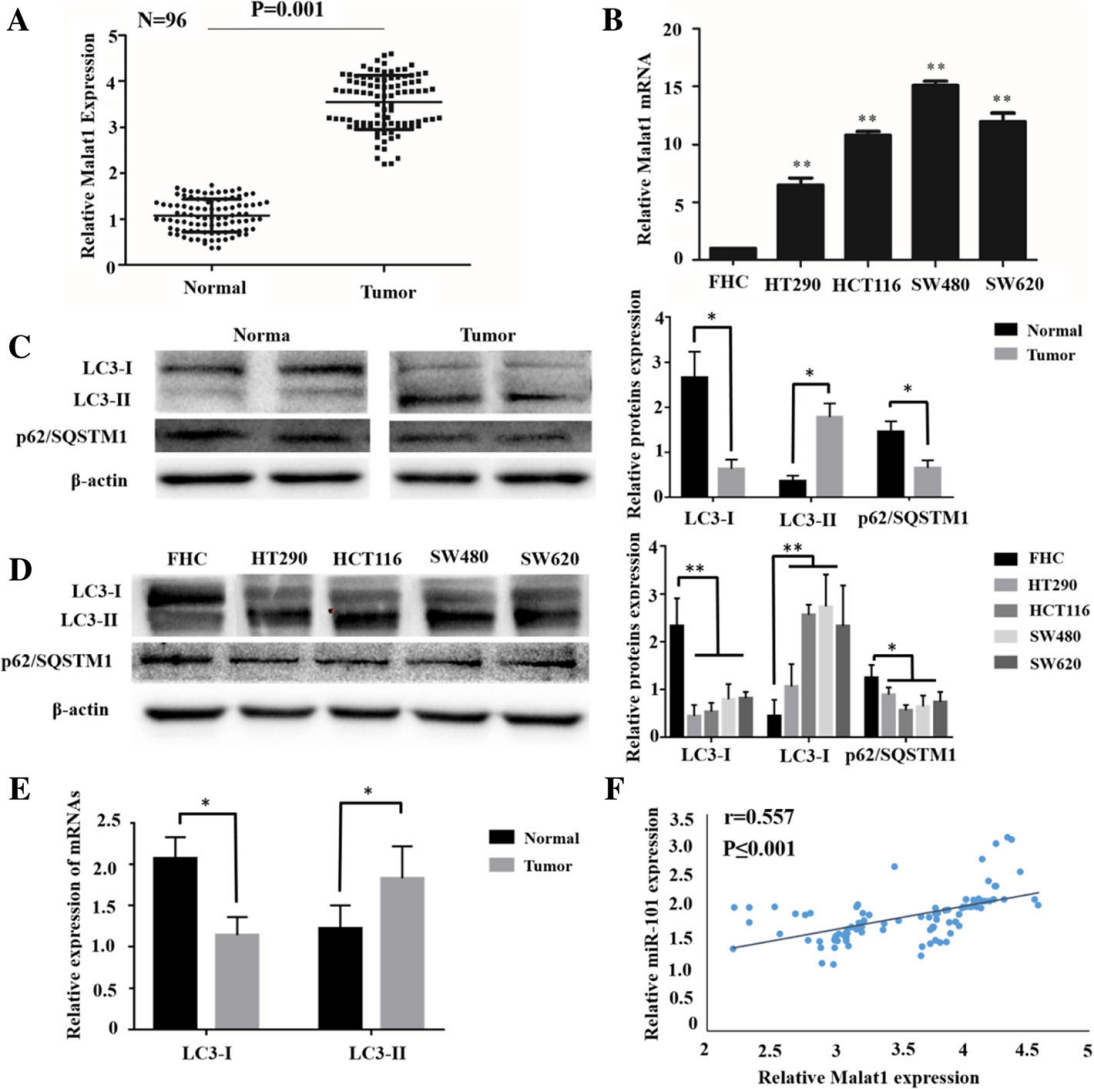
Malat1 is remarkably overexpressed in CRC, and associates with autophagy activation in CRC. A-B: qRT-PCR assay shows Malat1 expression in CRC tissues and adjacent normal tissues (**a**), 4 CRC cell lines and a human normal colorectal mucosa cell line FHC (**b**). HCT116 cell line and SW620 cell line were chosen to perform subsequent experiments. **c-d** Western blot shows that LC3-II/I and p62/SQSTM1 expression in CRC tissues and adjacent normal tissues (**a**), CRC cell lines and human normal colorectal mucosa cell line (**d**), the columns show the mean for three separate experiments. **e** qRT-PCR assay shows LC3-II/I expression in CRC tissues and adjacent normal tissues. **f** A significant positive correlation between Malat1 and LC3-II mRNA levels in CRC tissues. Bars, sd. *p < 0.05, **p < 0.01

Ninety-six pairs of CRC tissues and adjacent normal tissues were detected by qRT-PCR to reveal the role of Malat1 in CRC. When compared with adjacent normal tissues, the expression of Malat1 in CRC tissues was extremely high (*p* = 0.001; Fig. 1a). In addition, this study detected the expression level of Malat1 in 4 CRC cell lines – HCT290, HCT116, SW480 and SW620 – and the human normal colorectal mucosa cell line FHC, finding that Malat1 expression was remarkably high in CRC cells in comparison with FHC (Fig. 1b). In the meantime, this study detected the relationship between Malat1 and autophagy in CRC tissues and cell lines by the Western blot assay. As presented in Fig. 1c and d, LC3-II/I, which reflects autophagosome formation, was extremely increased in CRC tissues and cell lines compared with normal tissues and cells. Furthermore, it was found that the expression of p62/SQSTM1 and the polyubiquitin binding protein that reflected the activity of autophagy remarkably decreased in the CRC tissues and cells (Fig. 1c and d). In addition to this, the expression level of LC3–1 and LC3-II in CRC tissues was detected. As shown in Fig. 1e, compared with adjacent normal tissues, LC3-I was down-regulated in tumors (*p* < 0.05), while LC3-II was up-regulated (*p* < 0.05). Hence, a positive correlation was found in CRC tissues between Malat1 and LC3-II mRNA levels (Fig. 1f). Taken together, Malat1 was prominently up-expressed in CRC tissues, and was relevant to the increased autophagy activation in them.

### Malat1 increased cell proliferation and reduced apoptosis by activating autophagy

**Fig. 2:**
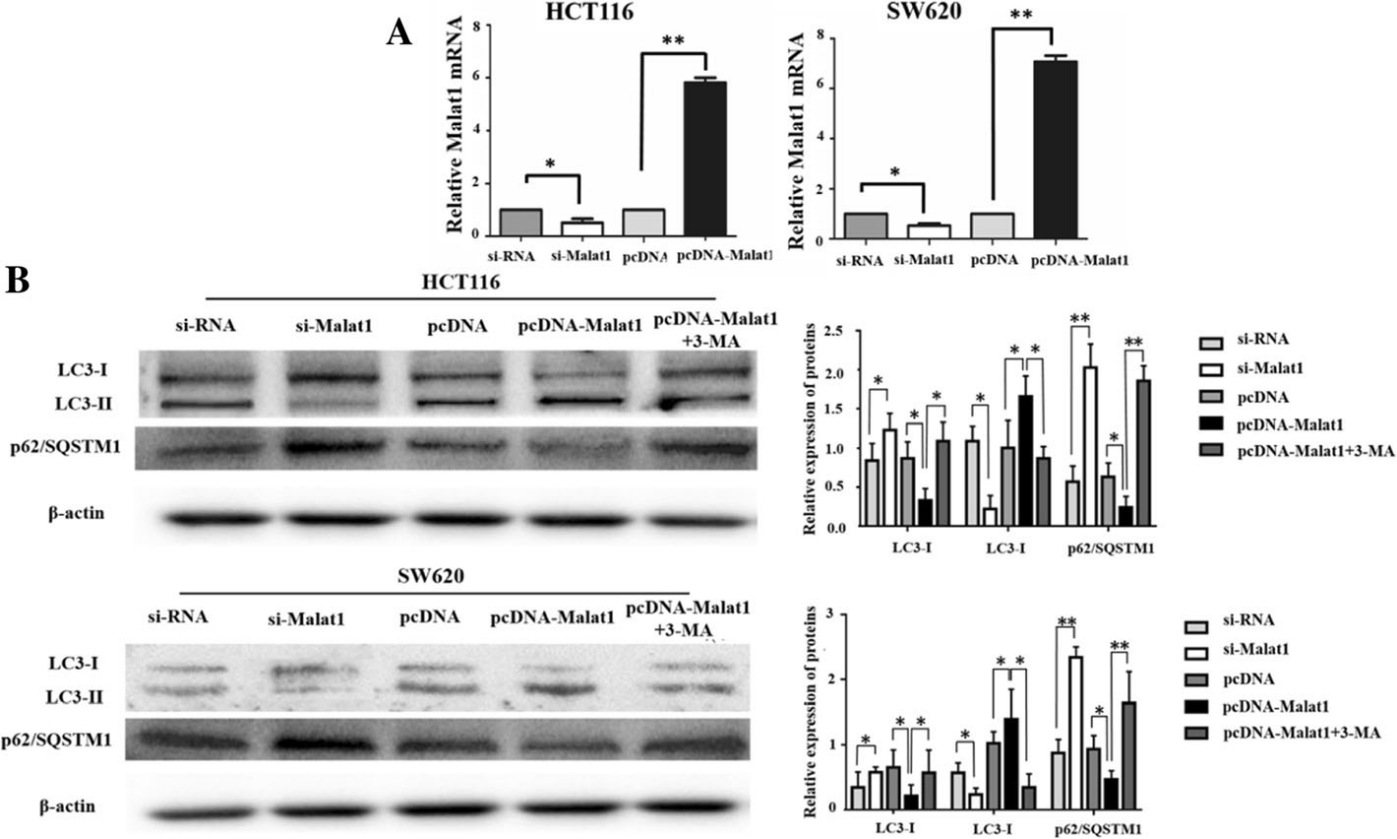
Abnormal expression of Malat1 activates autophagy in CRC. **a** qRT-PCR assay shows Malat1 level in HCT116 and SW620 cells transfected with si-RNA, si-Malat1, pcDNA, pcDNA-Malat1 or pcDNA-Malat1 + 3-MA. **b** Western blot assay shows the effects of Malat1 downregulation or upregulation on LC3-II/Iand p62/SQSTM1 expression in HCT116 and SW620 cells. The columns show the mean for three separate experiments. *p < 0.05, **p < 0.01

**Fig. 3:**
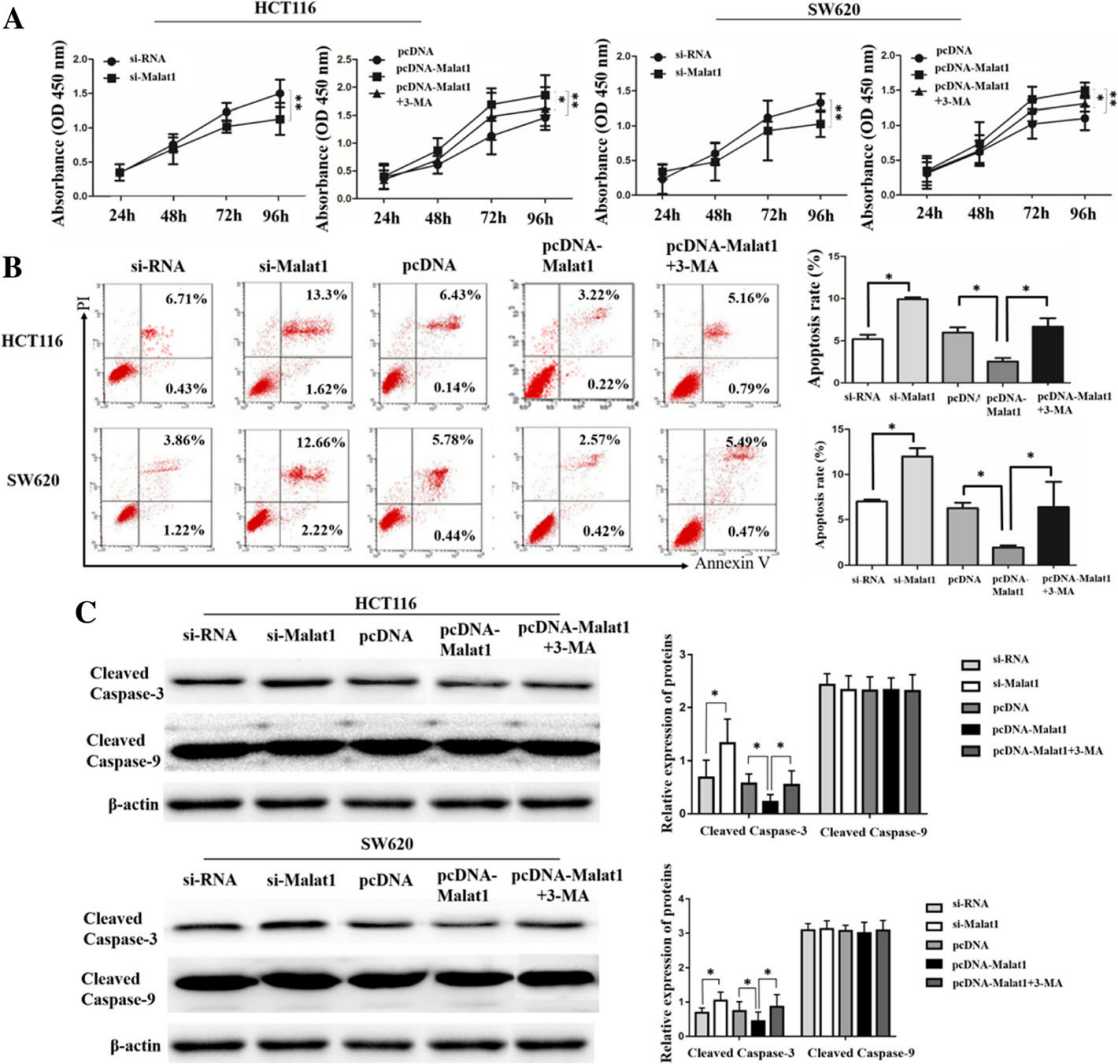
Malat1 increased cell proliferation and deduced apoptosis by activating autophagy. CCK-8 proliferation assay (**a**), flow cytometry (FCM) assay (**b**) and Western blot assay (**c**) show the effects of Malat1 downregulation or Malat1 upregulation on HCT116 and SW620 cell proliferation, apoptosis and apoptosis protein (cleaved caspase-3 and cleaved caspase-9) expression levels, and autophagy inhibition by 3-MA relieves induced cell proliferation and reduced cell apoptosis and cleaved caspase-3 expression level by Malat1 upregulation. The columns show the mean for three separate experiments. *p < 0.05, **p < 0.01

Due to the low transfection efficiency of other cell lines, HCT116 and SW620 cell lines were used in this experiment. To investigate the effect of Malat1 on autophagy in CRC cells, this study performed qRT-PCR and western blot assays in HCT116 and SW620 cells after transfection with si-RNA, si-Malat1, pcDNA, or pcDNA-Malat1. As presented in Fig. 2a, the expression level of Malat1 was extremely down-regulated by si-Malat1 transfection, yet was up-regulated by pcDNA-Malat1 transfection. Furthermore, the results in Fig. 2b indicated that down-regulation of Malat1 expression reduced the LC3-II/LC3-I level while increasing p62/SQSTM1 expression. Correspondingly, the up-regulation of Malat1 promoted the conversion of LC3-I to LC3-II while decreasing the expression of p62/SQSTM1 (Fig. 2b). To detect whether autophagy activated by Malat1 had been involved in cell proliferation and apoptosis, CCK8 proliferation assay was performed to detect the effects of Malat1 on the proliferation of HCT116 and SW620 cells. According to the results, a lower cell proliferation rate was found in the si-Malat1 group compared with the si-RNA group (Fig. 3a). Subsequently, this study focused on the proliferation rate of HCT116 and SW620 cells in the pcDNA group, Malat1 group, and Malat1 + 3-MA group. The results showed that cell proliferation could be promoted by Malat1 up-regulation, and this effect could be alleviated by autophagy inhibitor 3-MA (Fig. 3a). Furthermore, this study detected the role of Malat1 in CRC cell apoptosis by flow cytometry, indicating that the cell apoptosis rate in the si-Malat1 group was significantly higher than in the si-RNA group (Fig. 3b). Meanwhile, Malat1 up-regulation reduced the cell apoptosis rate and could be relieved by 3-MA through autophagy inhibition (Fig. 3b). To further investigate the apoptosis induced by Malat1, the expression of cleaved caspase-3 and cleaved caspase-9, as well as the proteins of apoptosis markers in the caspase protease family, were assessed. The expression of cleaved caspase-3 significantly increased in the cells down-regulated by Malat1 compared with the control transfected cells (*p* < 0.01; Fig. 3c). Likewise, the up-regulation of Malat1 resulted in the decrease of cleaved caspase-3 in comparison with the pcDNA group (*p* < 0.01; Fig. 3c). Surprisingly, the expression of cleaved caspase-9 in up-regulation Malat1 cells or down-regulation cells showed no difference when compared with the control transfected cells (Fig. 3c). These data suggested that by activating autophagy, Malat1 could increase cell proliferation and meanwhile inhibited apoptosis in CRC cells.

### Malat1 activated autophagy by sponging miR-101

**Fig. 4:**
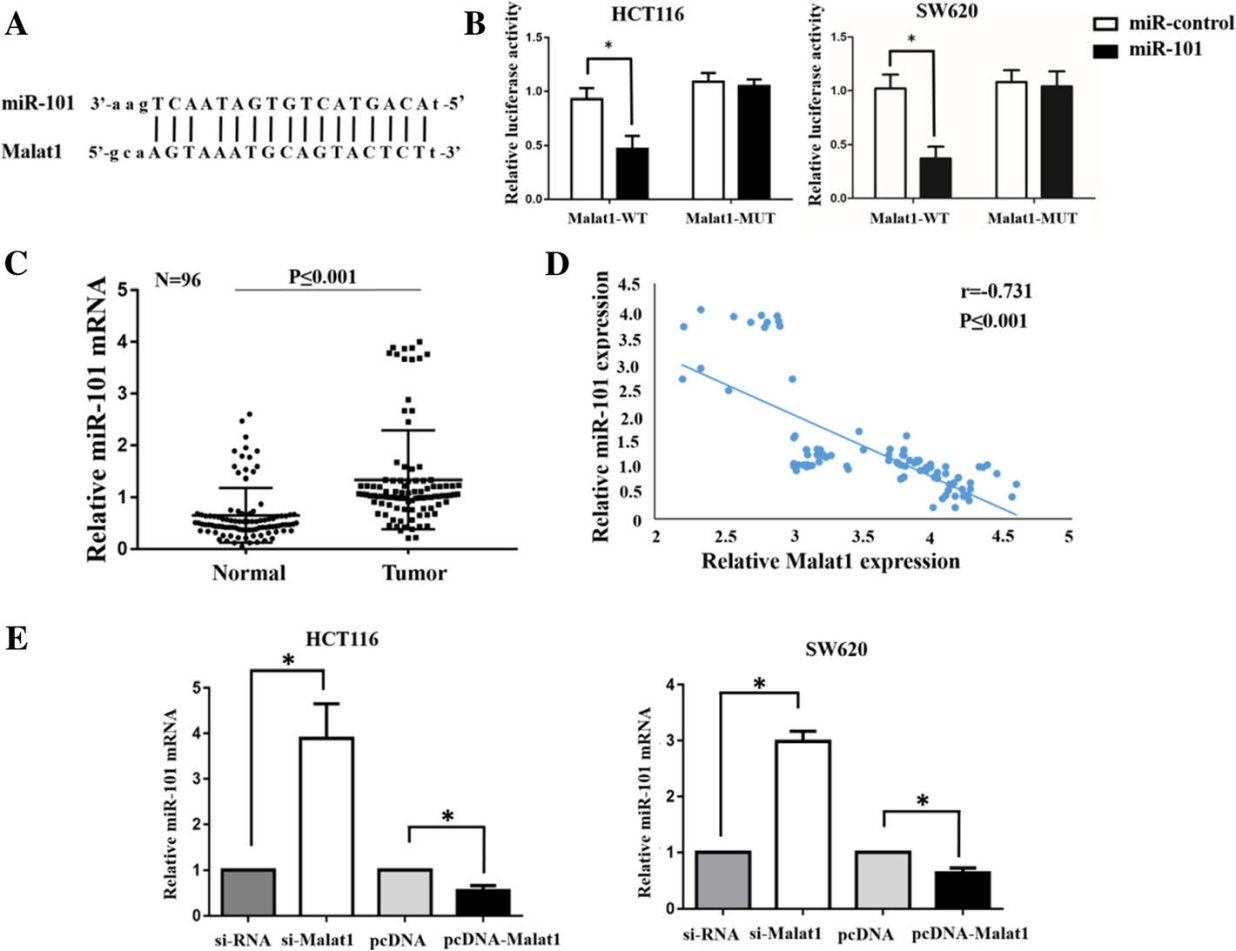
miR-101 works as a target RNA of Malat1. **a-b** Dual-luciferase reporter assay shows that co-transfection with miR-101 overexpression mimics significantly reduces luciferase activity of the reporter containing Malat1-WT, but it has less effect on the reporter containing Malat1-MUT in HCT116 and SW620 cells. **c** qRT-PCR assay shows miR-101 expression in CRC tissues and adjacent normal tissues. **d** An inverse correlation between Malat1 and miR-101 expression in CRC tissues. **e** qRT-PCR assay shows the effects of Malat1 down-regulation or up-regulation on miR-101 expression in HCT116 and SW620 cells. The columns show the mean for three separate experiments. Bars, sd. *p < 0.05, **p < 0.01

**Fig. 5:**
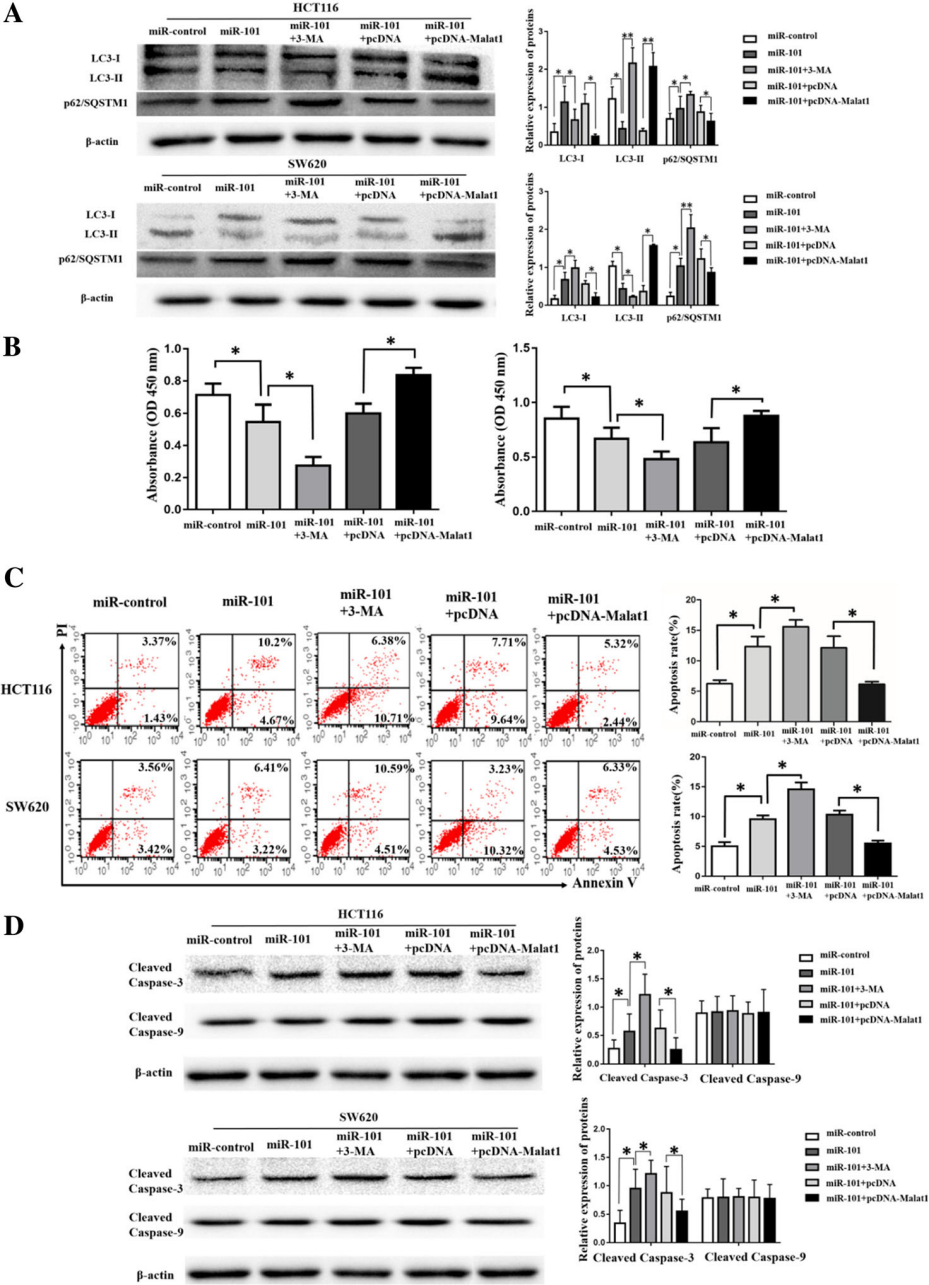
Rescue assay. Western blot (**a**), CCK-8 assays (**b**) , flow cytometry (FCM) assay (**c**) and western blot (**d**) show that the increase of miR-101 reduces LC3-II/I level and cell proliferation rate, and enhances p62/SQSTM1 expression, apoptosis rate and cleaved caspase-3 expression level, while the treatment of miR-101 + pcDNA-Malat1 abrogates effects induced by miR-101 increase in HCT116 and SW620 cells. The columns show the mean for three separate experiments. Bars, sd. *p < 0.05, **p < 0.01

It was identified that miR-101 was a target of Malat1 by binding to the complementary sequences in Fig. 4a [9]. To detect whether the function of Malat1 in autophagy, which influenced cell proliferation and apoptosis, was dependent on miR-101, the dual-luciferase reporter assay was performed. The luciferase activity of the Malat1-WT reporter gene was significantly restrained after co-transfection with miR-101 overexpression mimics (miR-101) when compared with the control (miR-control), while the luciferase activity of the Malat1-MUT reporter gene showed no significantly change (Fig. 4b). Furthermore, the expression level of miR-101 in CRC tissues was detected. As presented in Fig. 4c, compared with the adjacent normal tissues, the expression of miR-101 in CRC tissues was extremely high (*p* ≤ 0.001). Thereby, a negative relationship between Malat1 and miR-101 expression was observed in the CRC tissues (Fig. 4d). Moreover, the miR-101 expression was increased by Malat1 down-regulation, yet was decreased by Malat1 up-regulation (Fig. 4e). Subsequently, western blot, CCK-8 and apoptosis were also performed to detect the functions of Malat1 by targeting miR-101. As indicated in Fig. 5a-d, the overexpression of miR-101 inhibited the conversion of LC3–1 to LC3-II as well as cell proliferation rate, yet increased the p62/SQSTM1 expression, apoptosis rate and cleaved caspase-3 expression. Nonetheless, the co-expression of Malat1 with miR-101 could abrogate the effects induced by miR-101 overexpression. Surprisingly, the expression level of cleaved caspase-9 in up-regulated miR-101 cells or in miR-101 + pcDNA-Malat1 cells showed no difference when compared with the control transfected cells (Fig. 5d). These data suggested that Malat1 activated autophagy could promote cell proliferation and inhibit apoptosis by sponging miR-101 in CRC cells.

## Discussion

Malat1, as an oncogene, plays a crucial role in various tumors [18, 20, 21]. It has been demonstrated that Malat1 is over-expressed in CRC tissues, indicating a poor prognosis in CRC patients [17]. Nevertheless, there are rare reports regarding the mechanism of Malat1 participating in the tumorigenesis and development of CRC. Autophagy, which participates in cell regulation and intracellular homeostasis, is always identified as an evolutionarily conserved catabolic process [15]. It has been shown that autophagy is associated with poor outcome and is effective as a prognostic marker in CRC [22].Recently, an increasing number of studies have revealed that Malat1 promotes tumorigenesis by stimulating autophagy in many cancers [9, 15, 16]. For instance, Li L et al. determined that Malat1 inhibits autophagy in pancreatic cancer through interacting with HuR and the abnormal expression level of TIA-1 [15]. Gao D et al. found that Malat1 promoted autophagy in multiple myeloma through the up-regulation of HMGB1 in vitro and in vivo [14]. Nonetheless, the mechanism regarding the role of Malat1 in autophagy regulation in CRC remains unclear. This study confirmed that Malat1 was over-expressed in CRC tissues and cell lines, having a positive correlation with the LC3-II expression level in CRC. In addition, it was found for the first time that Malat1 promoted cell proliferation and decreased apoptosis through autophagy activation in CRC cell lines.

This study further determined the mechanisms by which Malat1 regulated autophagy in CRC cells. The well-known character of lncRNAs, as competitive endogenous RNAs (ceRNAs), could prevent the common miRNA binding to the ancestral gene [23]. YiRen H et al. discovered that Malat1 acted as a competing endogenous RNA for miR-23b-3p and attenuated the inhibitory effect of miR-23b-3p on ATG12, leading to the chemo-induced autophagy and chemoresistance in gastric cancer cells [24]. Fu Z et al. determined that Malat1, working as an endogenous sponge gene, reduced the miR-101 expression by binding to miR-101 directly in glioma [9]. Thereby, it was assumed that Malat1 accelerated autophagy activation by targeting the expression of miR-101. To confirm the prediction, a series of cell experiments were performed. As the results showed, autophagy and proliferation were inhibited by miR-101, whereas Malat1 abolished the effects induced by miR-101. Furthermore, a negative correlation was detected between the Malat1 and miR-101 in CRC. Taken together, the evidence showed that Malat1 promoted cell proliferation through activating autophagy and suppressing the miR-101 expression in the CRC cell lines.

Apoptosis, also termed programmed cell death, is an elaborate cellular homeostasis mechanism that ensures correct organ development, tissue remodeling, immune response, and tumor suppression. Cancer-associated defects in apoptosis play important roles in tumor pathogenesis. Defects in apoptosis also increased the threshold for cell death, thereby requiring higher doses for tumor killing [22]. Thus, the activation of apoptosis in tumor cells is a promising strategy for the treatment of cancer. Caspase is a crucial hallmark of the malignant degree of cancer [25]. It has been demonstrated that caspases exerts a significant effect on “self-eating” autophagy [26]. Moreover, caspases can turn off the autophagic response by degrading autophagy proteins (i.e., beclin-1, Atg5, and Atg7) after being activated by the pro-apoptosis signals [27]. In the meantime, activated caspases transformed the pro-autophagic proteins to pro-apoptotic proteins and triggered apoptotic cell death [27]. In this study, the relationship between the most representative apoptosis markers (cleaved caspase-3 and cleaved caspase-9 [28]) and Malat1 was detected, which was in agreement with the increased expression of cleaved caspase-3, while the expression of cleaved caspase-9, another caspase protease, indicated no significant difference in the CRC cell lines in comparison with the control. As is widely known, cleaved caspase-9 is the apoptotic initiator protease of the intrinsic or mitochondrial apoptotic pathway [28]. It was proposed in this study that apoptosis was not induced through Malat1-activated autophagy in the mitochondrial apoptotic pathway. Nonetheless, further experiments are needed to explore the mechanism of autophagy and apoptosis.

## Conclusion

This study revealed for the first time that Malat1 facilitated cell proliferation and decreased apoptosis through activating autophagy by miR-101 expression suppression in CRC cell lines. The above results provided a more in-depth understanding of tumorgenesis of CRC, as well as helping to find more effective treatments for colorectal cancer.

## Data Availability

The data used to support the findings of this study are available from the corresponding author upon request.

## Abbreviations

3-MA: 3-methyladenine
CRC: Colorectal cancer
FCM: Flow cytometry
LNC: Long non-coding RNA
Malat1: Metastasis-associated lung adenocarcinoma transcript 1
qRT-PCR: quantitative real-time PCR

## References

[1] Esteller M (2011). Non-coding RNAs in human disease. Nat Rev Genet.

[2] Quinn JJ, Chang HY (2016). Unique features of long non-coding RNA biogenesis and function. Nat Rev Genet.

[3] Guil S, Esteller M (2012). Cis-acting noncoding RNAs: friends and foes. Nat Struct Mol Biol.

[4] Kornienko AE, Guenzl PM, Barlow DP, Pauler FM (2013). Gene regulation by the act of long non-coding RNA transcription. BMC Biol.

[5] Tomasoni S, Benigni A (2013). Post-transcriptional gene regulation makes things clearer in renal fibrosis. J Am Soc Nephrol.

[6] Geisler S, Coller J (2013). RNA in unexpected places: long non-coding RNA functions in diverse cellular contexts. Nat Rev Mol Cell Biol.

[7] Yuan N, Zhang G, Bie F, Ma M, Ma Y, Jiang X (2017). Integrative analysis of lncRNAs and miRNAs with coding RNAs associated with ceRNA crosstalk network in triple negative breast cancer. Onco Targets Ther.

[8] Yang L, Wang H, Shen Q, Feng L, Jin H (2017). Long non-coding RNAs involved in autophagy regulation. Cell Death Dis.

[9] Fu Z, Luo W, Wang J, Peng T, Sun G, Shi J (2017). Malat1 activates autophagy and promotes cell proliferation by sponging miR-101 and upregulating STMN1, RAB5A and ATG4D expression in glioma. Biochem Biophys Res Commun.

[10] Gutschner T, Hammerle M, Diederichs S (2013). MALAT1 -- a paradigm for long noncoding RNA function in cancer. J Mol Med.

[11] Klionsky DJ, Emr SD (2000). Autophagy as a regulated pathway of cellular degradation. Science.

[12] Rebecca VW, Amaravadi RK (2016). Emerging strategies to effectively target autophagy in cancer. Oncogene.

[13] Yang L, Zhang X, Li H, Liu J (2016). The long noncoding RNA HOTAIR activates autophagy by upregulating ATG3 and ATG7 in hepatocellular carcinoma. Mol BioSyst.

[14] Gao D, Lv AE, Li HP, Han DH, Zhang YP (2017). LncRNA MALAT-1 elevates HMGB1 to promote autophagy resulting in inhibition of tumor cell apoptosis in multiple myeloma. J Cell Biochem.

[15] Li L, Chen H, Gao Y, Wang YW, Zhang GQ, Pan SH (2016). Long noncoding RNA MALAT1 promotes aggressive pancreatic Cancer proliferation and metastasis via the stimulation of autophagy. Mol Cancer Ther.

[16] Yuan P, Cao W, Zang Q, Li G, Guo X, Fan J (2016). The HIF-2alpha-MALAT1-miR-216b axis regulates multi-drug resistance of hepatocellular carcinoma cells via modulating autophagy. Biochem Biophys Res Commun.

[17] Zheng HT, Shi DB, Wang YW, Li XX, Xu Y, Tripathi P (2014). High expression of lncRNA MALAT1 suggests a biomarker of poor prognosis in colorectal cancer. Int J Clin Exp Pathol.

[18] Gutschner T, Hammerle M, Eissmann M, Hsu J, Kim Y, Hung G (2013). The noncoding RNA MALAT1 is a critical regulator of the metastasis phenotype of lung cancer cells. Cancer Res.

[19] Seglen PO, Gordon PB (1982). 3-Methyladenine: specific inhibitor of autophagic/lysosomal protein degradation in isolated rat hepatocytes. Proc Natl Acad Sci U S A.

[20] Liu JH, Chen G, Dang YW, Li CJ, Luo DZ (2014). Expression and prognostic significance of lncRNA MALAT1 in pancreatic cancer tissues. Asian Pac J Cancer Prev.

[21] Fan Y, Shen B, Tan M, Mu X, Qin Y, Zhang F (2014). TGF-beta-induced upregulation of malat1 promotes bladder cancer metastasis by associating with suz12. Clin Cancer Res.

[22] Sun J, Zhang C, Bao YL, Wu Y, Chen ZL, Yu CL (2014). Parthenolide-induced apoptosis, autophagy and suppression of proliferation in HepG2 cells. Asian Pac J Cancer Prev.

[23] Tay Y, Kats L, Salmena L, Weiss D, Tan SM, Ala U (2011). Coding-independent regulation of the tumor suppressor PTEN by competing endogenous mRNAs. Cell.

[24] Lorenzen JM, Thum T (2016). Long noncoding RNAs in kidney and cardiovascular diseases. Nat Rev Nephrol.

[25] Su Z, Yang Z, Xu Y, Chen Y, Yu Q (2015). Apoptosis, autophagy, necroptosis, and cancer metastasis. Mol Cancer.

[26] Stupack DG (2013). Caspase-8 as a therapeutic target in cancer. Cancer Lett.

[27] Wu H, Che X, Zheng Q, Wu A, Pan K, Shao A (2014). Caspases: a molecular switch node in the crosstalk between autophagy and apoptosis. Int J Biol Sci.

[28] Shalini S, Dorstyn L, Dawar S, Kumar S (2015). Old, new and emerging functions of caspases. Cell Death Differ.

